# Foulant Characteristics Comparison in Recycling Cooling Water System Makeup by Municipal Reclaimed Water and Surface Water in Power Plant

**DOI:** 10.1155/2015/746064

**Published:** 2015-03-29

**Authors:** Xu Ping, Wang Jing, Zhang Yajun, Wang Jie, Si Shuai

**Affiliations:** ^1^Key Laboratory of Urban Stormwater System and Water Environment, Ministry of Education, Beijing University of Civil Engineering and Architecture, Beijing 100044, China; ^2^Beijing Datang Real Investment Center, Beijing 100191, China

## Abstract

Due to water shortage, municipal reclaimed water rather than surface water was replenished into recycling cooling water system in power plants in some cities in China. In order to understand the effects of the measure on carbon steel corrosion, characteristics of two kinds of foulant produced in different systems were studied in the paper. Differences between municipal reclaimed water and surface water were analyzed firstly. Then, the weight and the morphology of two kinds of foulant were compared. Moreover, other characteristics including the total number of bacteria, sulfate reducing bacteria, iron bacteria, extracellular polymeric substance (EPS), protein (PN), and polysaccharide (PS) in foulant were analyzed. Based on results, it could be concluded that microbial and corrosive risk would be increased when the system replenished by municipal reclaimed water instead of surface water.

## 1. Introduction

Per capita water resources of China are only a quarter of average value of the world. China was belonging to one of 13 countries with an extreme deficiency of water resources in the world [1]. At the same time, rapid economic development and serious environment pollution exacerbate the situation of water shortage. Problems caused by water shortage have already become the bottleneck of economic and social sustainable development in China. So municipal reclaimed water rather than surface water was replenished into recycling cooling water system in power plants in some severe water shortage cities, such as Beijing, Tianjin, Taiyuan, Qingdao, and Xi'an [2]. Compared with surface water, municipal reclaimed water contains more microorganisms and higher content of inorganics, organics, nitrogen, and phosphorus. Recycling cooling water system would face more microbial and corrosive problems with the system makeup by municipal reclaimed water instead of surface water.

In fact, biofilm growth, pipe corrosion, and water quality deterioration have been found in the real system [3, 4].

Using surface water to replenish recycling cooling water system has been studied systematically. Many works are concentrated in the chemical stability, corrosion, and scaling mechanism, corresponding to control technology [5–8]. The existing studies provide important theoretical and technical support for the system security. However, water quality of municipal reclaimed water is more complicated than that of surface water. So more biochemical, physical, and chemical reactions will happen in the recycling cooling water system when made up by municipal reclaimed water instead of surface water, which will result in more foulants and severe metal corrosion on the interface of pipe. At present, fewer reports about foulant, metal corrosion, and microorganism in the recycling cooling water system makeup by municipal reclaimed water are found.

Two kinds of foulants on the carbon steel coupons in the system makeup by either municipal reclaimed water or surface water were studied, respectively, by static experiment in the paper. Firstly, 23 water quality indexes of municipal reclaimed water and surface water were comprehensively analyzed by chemical methods. Then foulant characteristics of both systems, mainly including the total number of bacteria, sulfate reducing bacteria, iron bacteria, extracellular polymeric substance (EPS), protein (PN), polysaccharide (PS), and weight of foulant and its morphology, were analyzed by microbiological methods and scanning electron microscopy (SEM) technology. Based on experimental results, the effects of different makeup water resources on foulants in recycling cooling water system were discussed.

## 2. Materials and Methods

**Figure 1:**
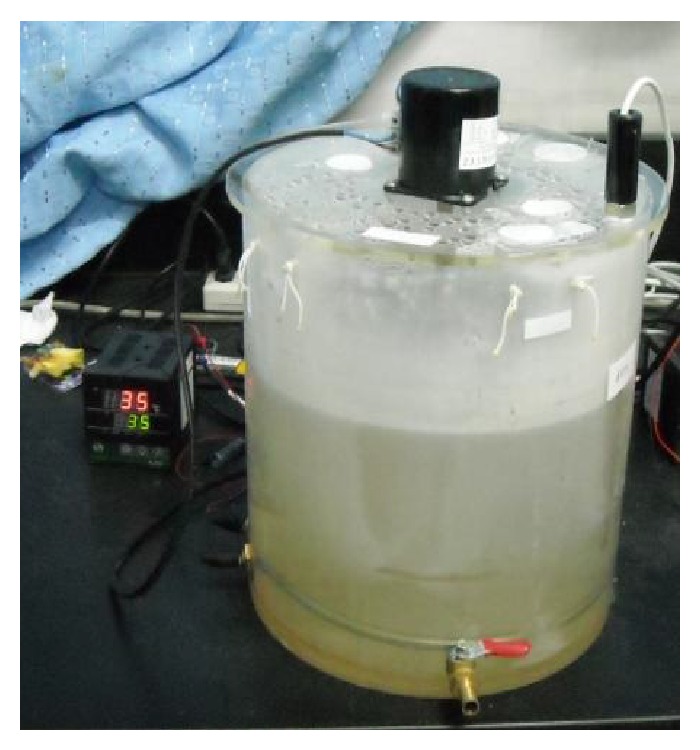
Static reactor.

Two groups of parallel artificial reactors were set, as shown in Figure 1. After 20^#^ carbon steel coupons were hanged, 10 L municipal reclaimed water and 10 L surface water were added into the reactors, respectively. Then temperatures, flow velocities, and concentration ratios of the reactors were set to 35°C, 0.7 m/s, and 1.0 times. Running time was 50 days. During the experiment, national type I carbon steel coupons were adopted. Size of the coupon was 5 cm × 2.5 cm × 0.2 cm. Surface water was taken from Beijing Jingmi diversion channel (Linglong Lu sections). Municipal reclaimed water was taken from municipal reclaimed water pipeline of one thermal power plant in Beijing.

### 2.1. Analytical Methods of Water Quality

The national standard analytical methods for water quality were adopted in the experiment, as shown in Table 1.

### 2.2. Analytical Methods of Microorganism in Foulant

#### 2.2.1. The Total Number of Bacteria in Foulant

Rapid microbial fluorescence detector Pi-102 produced by American Hygiene Company was employed for total number of bacteria analysis. Principle of the instrument is that ATP was released from the microbial cells through the cell lysis reagent extractant. Then luciferase reaction agent, bioluminescence reagent, was added and fluorescence was generated due to the reaction between bioluminescence reagent and ATP. At last, the total number of bacteria was determined according to testing fluorescence value.

#### 2.2.2. Sulfate Reducing Bacteria and Iron Bacteria in Foulant

Sulfate reducing bacteria (SRB) were analyzed by MPN method according to “Examination of bacteria and algae in industrial circulating cooling water—Part 5: Examination of sulfate-reducing bacteria” (GB/T14643.5 2009).

Iron bacteria were analyzed by MPN method according to “Examination of bacteria and algae in industrial circulating cooling water—Part 6: Examination of iron bacteria” (GB/T 14643.6 2009).

#### 2.2.3. EPS, PN, and PS in Foulant

Peel off foulant from coupons and put it into a beaker. Add 50 mL buffer solution (2 mmol/L Na_3_PO_4_, 4 mmol/L NaH_2_PO_4_, 9 mmol/L NaCl, and 1 mmol/L KCl, pH = 10) into the beaker. Then dip 2 to 5 cotton swabs into the buffer solution and use them to scrub coupons back and forth. Put the swabs into the beaker also. Vibrate the beaker by ultrasonic apparatus for 10 min. Add 0.3 mL formaldehyde into the beaker and stir the beaker 0.5 h at 300 r/min. After adding 50 mL 0.04 mol/L NaOH solution into the beaker, stir it for 1 h and centrifuge it for 10~20 min. Filtrate supernatant fluid by 0.45 *μ*m microporous membrane and take clear liquid for experiment.

**Figure 2:**
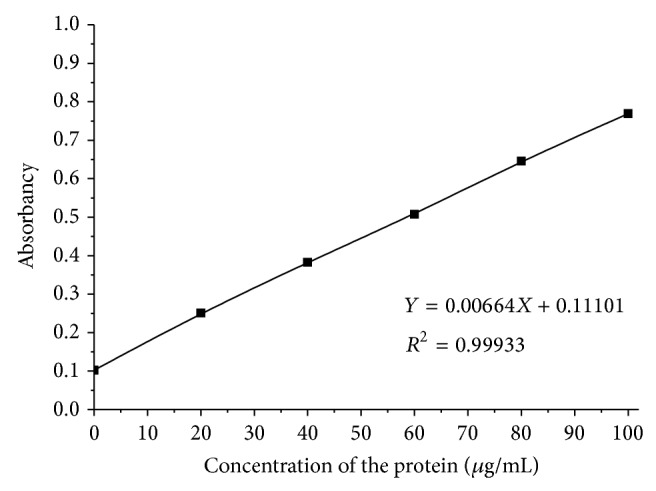
Protein standard curve.

**Figure 3:**
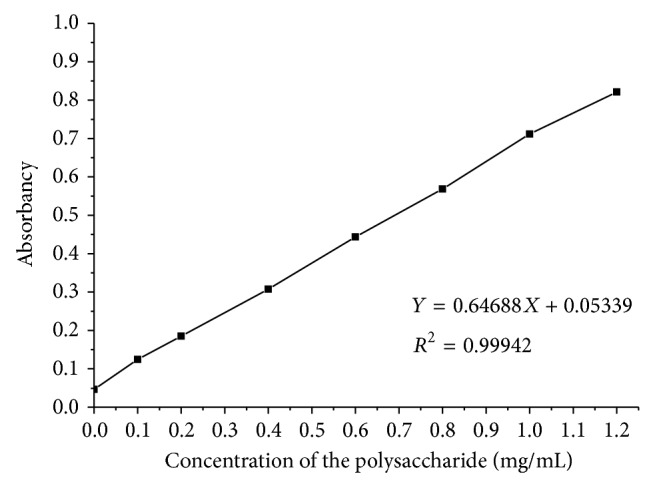
Polysaccharide standard curve.

Protein was tested by coomassie brilliant blue G-250 method. Standard curve of protein in the experiment was shown in Figure 2. Polysaccharide was tested by sulfuric acid, phenol method which was most widely used. Standard curve of polysaccharide was shown in Figure 3. EPS is mainly composed of protein and polysaccharide.

## 3. Results and Discussion

### 3.1. Water Quality of Municipal Reclaimed Water and Surface Water

Comparison of water quality of municipal reclaimed water and surface water was shown in Table 2.

According to Table 2, except aluminum and copper, the concentrations of turbidity, pH, alkalinity, zinc, manganese, total iron, and bicarbonate in municipal reclaimed water were lower than those in surface water, but the concentrations of conductivity, TDS, chloride ion, sulfate, calcium hardness, COD_Mn_, total hardness, total phosphorus, soluble phosphorus, orthophosphate, silicon, surfactant, and total bacteria were higher. The concentrations of conductivity, TDS, chloride ion, silicon, and surfactant in municipal reclaimed water were, respectively, 1.5~2 times, 1.6~1.9 times, 1.9~4.5 times, 2.9 times, and 2.8 times higher than those in surface water. The concentrations of COD_Mn_, total phosphorus, soluble phosphorus, orthophosphate, and total nitrogen were about 1.5~2 times higher than those in surface water. The total number of bacteria in municipal reclaimed water was one order of magnitude higher than that in surface water.

Compared with surface water, more sulfate, calcium, phosphate, and silica in municipal reclaimed water would increase scale risks in the recycling cooling water system. However, higher anionic surfactant would play an opposite role. By comprehensive analysis according to Table 2, the concentration of surfactant in municipal reclaimed water was 2.8 times higher than that in surface water. Because the difference of surfactants in municipal reclaimed water and surface water was bigger than that of sulfate, phosphate, and silica, scaling risk would reduce with the system makeup by municipal reclaimed water instead of surface water.

Chloride ion, sulfate, orthophosphate, and pH were the key indices for corrosion. Radius of chlorine ion is small and therefore that leads to its strong penetrability or adsorption ability. Chlorine ion could destroy the structure of oxide film on the metal and result in metal into active corrosion state from passive corrosion state. Although radius of sulfate ion was of larger and lower penetrability through passivation film than chloride ion, sulfate would be reduced to hydrogen sulfide by sulfate reducing bacteria and result in low pH nearby. So the pitting corrosion of metals happened. On the contrary, phosphate was beneficial to passivation of the metal surface. Main ingredient of the passivation membrane was *γ*-Fe_2_O_3_, which could delay the metal corrosion. Due to high chlorine ion and sulfate and low pH in municipal reclaimed water, risk of metal corrosion would increase in recycling cooling water system made up by municipal reclaimed water.

There were sunshine, high dissolved oxygen, and suitable temperature in recycling cooling water system, which were beneficial to microorganism growth. Moreover, high COD_Mn_, total phosphorus, orthophosphate total nitrogen, and the total number of bacteria in municipal reclaimed water would also increase microbial risk in recycling cooling water system.

### 3.2. Foulants in Recycling Cooling Water System Makeup by Municipal Reclaimed Water and Surface Water

**Figure 4:**
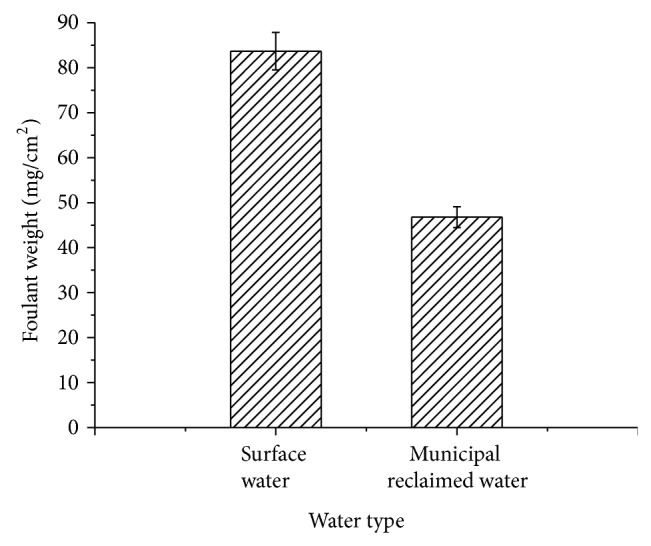
Weights of foulant in recycling cooling water system makeup by municipal reclaimed water and surface water. Note: unit of weight of foulant was wet weight of foulant per unit area of coupon.

Weight of foulant in recycling cooling water system makeup by municipal reclaimed water and surface water was shown in Figure 4.

**Figure 5:**
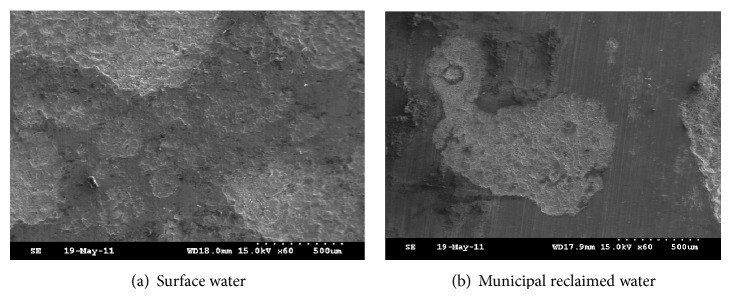
Morphology of foulants in recycling cooling water system makeup by municipal reclaimed water and surface water by SEM.

According to Figure 4, weight of foulant on the coupon in the system makeup by municipal reclaimed water was 46.8 mg/cm^2^, which was 0.56 times higher than that by surface water. SEM morphology in Figure 5 also clearly showed the differences of the foulants. According to Figure 5, it was clear that the surface of carbon steel coupon in the system makeup by surface water was almost entirely covered by foulant, whereas that by municipal reclaimed water was only covered partly.

**Figure 6:**
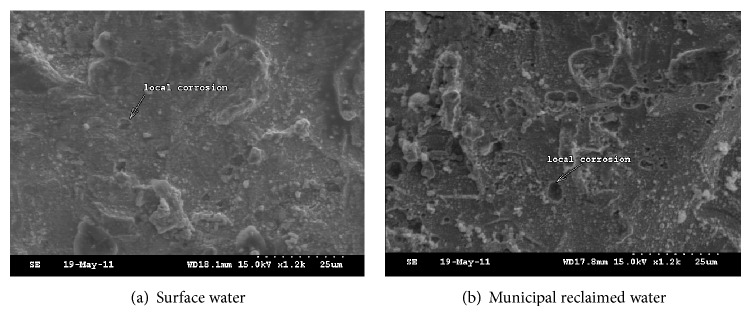
Corrosion morphology comparison in recycling cooling water system makeup by municipal reclaimed water and surface water.

Foulant in recycling cooling water system in power plant mainly formed by crystallization, particle, corrosion, and microorganism. The weight of foulant is mainly composed of the first three kinds of foulant. According to results of Section 3.1, crystallization foulant would be relieved in recycling cooling water system under the role of anionic surfactant in municipal reclaimed water, which was the main reason for the foulant weight decrease. Meanwhile, chloride ion in municipal reclaimed water was 2.5~4.5 times higher than that in surface water. Sulfate in municipal reclaimed water was also higher. So pitting corrosion of carbon steel was more likely to happen. Images by SEM confirmed the judgments above. According to Figure 6, carbon steel's corrosion in the system makeup by municipal reclaimed water belonged to pitting corrosion, which produced fewer foulants but more and deeper corrosion pits. By contrast, for carbon steel coupon in that by surface water, its corrosion surface was relatively uniform. The weight of corrosion foulant was more, but the depth of corrosion pit was relatively lighter.

In addition, compared with surface water, low turbidity in municipal reclaimed water would result in fewer particle foulants in recycling cooling water system.

Based on the above results, when municipal reclaimed water took the place of surface water to replenish into recycling cooling water system, the amount of foulant would be lower due to reduction of crystallization, corrosion, and particle deposition, which would lead to decrease of heat transfer resistance and energy losses of the heating equipment.

### 3.3. EPS, PN, and PS in Foulants in Recycling Cooling Water System Makeup by Municipal Reclaimed Water and Surface Water

**Figure 7:**
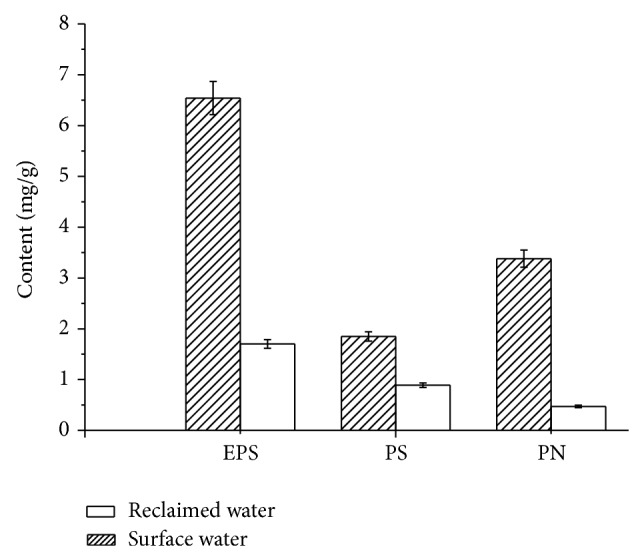
Comparison of EPS, PS, and PN in foulants in recycling cooling water system makeup by municipal reclaimed water and surface water.

Comparison of EPS, PS, and PN in foulants in recycling cooling water system makeup by municipal reclaimed water and surface water was shown in Figure 7.

According to Figure 7, EPS in unit mass of foulant in the system makeup by municipal reclaimed water was 1.70 mg/g. The value in that by surface water was 6.54 mg/g, which was 4 times higher compared with that by municipal reclaimed water. In the system makeup by municipal reclaimed water, contents of PN and PS in unit mass of foulant were, respectively, 0.47 mg/g and 0.89 mg/g, in which the ratios in EPS were corresponding to 27.6% and 52.4%. The proportion of PS was an absolute advantage. On the contrary, in the system makeup by surface water, the proportion of PN in EPS was up to 51.7% and the ratio of PS was relatively lower.

EPS was one kind of high-molecular compound which was secreted by microorganism and surrounded outside of the cell wall under certain environmental conditions. EPS mainly came from microbial metabolism, cell autolysis, deciduous of cell material, and influent water matrix [9]. By studying EPS of 27 kinds of heterotrophic bacteria, Tsuneda et al. found that PS and PN account for 75%~89% of EPS [10]. PN mainly came from intracellular secretion or substance release by cell digestion, which concluded many hydrophobic functional groups with a positive charge, such as alanine, cysteine, valine, and amino acid. According to the principle of thermal dynamics, the increase of hydrophobicity was helpful to reduce Gibbs energy on the surface of foulant [11]. Hydrophobicity played a key role in the initial stages of fouling formation [10, 12–17]. PS was synthesized outside of the cell. It consisted of hexose and pentose polymerization of polysaccharide (10~30 kda). Its chemical structure was highly branched and included many hydrophilic functional groups with negative charge, such as hydroxyl, carboxyl, and phosphate. Molecules on the chain wrapped around mutually. Thus, an amorphous network structure formed, which played a role of adsorption and flocculation by adsorption bridging [15]. Some scholars had put forward that acidic PS plays a role of bridge support for microbial aggregation and its strong three-dimensional structure by ionic bonds, neutral polysaccharide, and hydrogen bonding [18, 19]. This would be beneficial to the survival of microorganism in complex environment.

The content and composition of EPS were related to microbial species, growth status, and the types of substrate. In low organic matter and nutrient environment, available substrate by bacteria was less and its proliferation rate was low. EPS mainly came from cell autolysis and deciduous of cell material [20]. In high organic matter and nutrient conditions, microbial cell cannot put the whole carbon source for cell synthesis; excess carbon source was converted into EPS and extracellular polymeric substances were accumulated in EPS. EPS mainly came from the process of synthetic cell metabolism [21, 22]. Both municipal reclaimed water and surface water were poor nutrition environments. Microbial death and cell autolysis were the main source of EPS in the foulant. According to Table 2, the concentrations of COD_Mn_, total phosphorus, soluble total phosphorus, orthophosphate, and total nitrogen in municipal reclaimed water were 1.5~2 times higher than those in surface water. So metabolism of microorganism was of more activity. Results of Section 3.2 showed that the contents of crystallization, corrosion, and particulate depositions in the system makeup by municipal reclaimed water were less than those by surface water, which reduced the mass transfer resistance of organic matter, nutrients, and dissolved oxygen in foulant. At the same time, microbial assimilation was enhanced. On the whole, there were stronger microbial anabolic activity and lower death and cell autolysis rate in the system makeup by municipal reclaimed water than those in the surface water, which was the major cause of results of EPS decrease in foulant. Moreover, protein mainly came from intracellular secretion or substance release by cell digestion. EPS decreased when microbe died or cell autolysis ratio was low.

Components of EPS instead of its amount played greater role in foulant. Polyvalent metal ion, suspended organic matter, and inorganic matter in water were adsorbed by electrostatic interactions of protein in EPS. PN also could promote cell adhesion and cross linking structure formation. Higher proportion of PN in EPS would cause stronger hydrophobicity, higher cross linking degree, and more compact structure of foulant [23–27]. PS would promote the flocculation by reducing the effective critical potential of the bacterial cells due to cell walls wrapped by it. Higher proportion of PS would cause stronger flocculation effect and more microbial foulant. Lower PN and higher PS in the system makeup by municipal reclaimed water than that by surface water meant more microbial foulant and low compactness of foulant in the system.

### 3.4. Total Number of Bacteria, Iron Bacteria, and SRB in Foulants in Recycling Cooling Water System Makeup by Municipal Reclaimed Water and Surface Water

**Figure 8:**
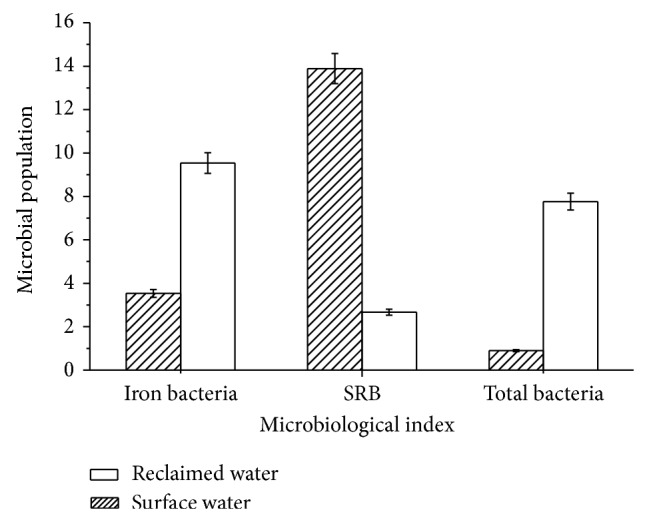
Microorganism comparisons of foulant in recycling cooling water system makeup by municipal reclaimed water and surface water. Note: the unit of the total number of bacteria was 10^6^ cfu/g; unit of SRB and iron bacteria was 10^3^ cfu/g.

Comparisons of the total number of bacteria, iron bacteria, and SRB in the foulant in recycling cooling water system makeup by municipal reclaimed water and surface water were shown in Figure 8.

According to Figure 8, total numbers of bacteria and iron bacteria in the system makeup by municipal reclaimed water were, respectively, 8.64 times and 8.64 times higher than those by surface water, but SRB was 80.8% lower.

In poor nutrition environment, microbial growth rate was proportional to nutrient metabolic rate. The higher the nutrient concentration, the greater the microbial growth rate. According to Table 2, the contents of COD_Mn_, total phosphorus, soluble total phosphorus, orthophosphate, and total nitrogen in municipal reclaimed water were 1.5~2 times higher than those in surface water, which was the main reason for the higher total number of bacteria and iron bacteria in the system. Moreover, based on results in Sections 3.2 and 3.3, in recycling cooling water system, less foulant weight and looser foulant structure were found if reclaimed water was used as makeup water instead of surface water. So transfer resistance decreased and dissolved oxygen increased in the foulant when the system is made up by reclaimed water instead of surface water. It was harmful to SRB in foulant. This was the main cause of number of SRB relatively being lower.

More microorganisms in municipal reclaimed water would increase biofilm and microbial foulant formation in the recycling cooling water system. The conclusion was in accordance with polysaccharide experimental results of Section 3.3.

Iron bacteria and SRB were typical corrosive microbes in recycling cooling water system. Iron bacteria belonged to aerobic bacteria, which could oxidize Fe^2+^ to Fe^3+^ and produce iron oxide precipitation on the metal surface. Moreover, precipitation on the surface also led to the formation of oxygen concentration cell and thus resulted in metal corrosion accelerated. SRB was anaerobic microbe. It used lactic acid or pyruvic acid as electron donor and sulfate as electron acceptor. By redox reaction, sulfate was reduced into bivalent sulfur and corrosion products were generated. In addition, role of cathodes depolarization was also the main reason of SRB corrosion. High iron bacteria and low SRB in municipal reclaimed water meant that metal corrosion would mainly be influenced by iron bacteria when recycling cooling water system replenished by reclaimed water.

Above all, in recycling cooling water system makeup by municipal reclaimed water instead of surface water, there were more numbers of microorganisms, high foulant viscosity, and more serious aerobic microbial corrosion.

## 4. Conclusion

Based on comparison of municipal reclaimed water and surface water, amount of foulant, the total number of bacteria, iron bacteria, SRB, EPS, PN, and PS in foulant on the carbon steel in recycling cooling water system makeup by municipal reclaimed water and surface water were studied, respectively, by microbiological methods and SEM technology. Main conclusions were shown as follows.

1. According to analysis results of 23 water quality indexes, compared with that in surface water, concentrations of conductivity, TDS, chloride ion, and surfactant in municipal reclaimed water were 2~4 times higher, concentrations of COD_Mn_, total phosphorus, soluble total phosphorus, orthophosphate, and total nitrogen were 1.5~2 times higher, and the total number of bacteria was one order of magnitude higher. The results showed that lower scaling, higher corrosion risk, and microbial risk would happen in the recycling cooling water system when it was replenished by municipal reclaimed water instead of surface water.
2. The weight of foulant in the recycling cooling water system makeup by municipal reclaimed water was 44% lower than that by surface water, but the total numbers of bacteria and iron bacteria in unit mass foulant were, respectively, 8.6 times and 2.7 times higher. The results indicated that heat transfer resistance and energy losses of the heating equipment would decrease but microbial corrosion would increase in the recycling cooling water system when it was replenished by municipal reclaimed water instead of surface water.
3. EPS in foulant in the recycling cooling water system makeup by municipal reclaimed water was 74% lower than that by surface water. Proportions of PN and PS in EPS were, respectively, 27.6% and 52.4% in the system makeup by municipal reclaimed water. The proportion of PS was an absolute advantage. On the contrary, in the system makeup by surface water, PN ratio in EPS was up to 51.7%; content of PS was relatively lower. The results revealed more microbial foulant and looser foulant structure in the recycling cooling water system when it was replenished by municipal reclaimed water instead of surface water.
4. Results of SEM showed that thicker foulant and uniform corrosion appeared in the recycling cooling water system makeup by surface water. When the system was replenished by municipal reclaimed water instead of surface water, the amount of foulant became less and metal pitting corrosion was more serious.

## Figures and Tables

**Table 1:** Analytical methods of water quality.

Number	Test index	The test method or instrument
1	pH	Glass electrode method
2	Turbidity	WZS-180 turbidity meter
3	Conductivity	DDSJ-308A conductivity meter
4	TDS	DDSJ-308A conductivity meter
5	Cl^−^	The Mol method (GB/T 15453-95)
6	SO_4_^2−^	Gravimetric method
7	Alkalinity	Acid-base indicator titration method (GB/T 15451-95)
8	Hardness	EDTA titrimetry (GB/T 15452-95)
9	HCO_3_^2−^	Titrimetry
10	COD	Permanganate titration (GB/T 15456-95)
11	TP	Ammonium molybdate spectrophotometer method (B G76 002-90)
12	Orthophosphate	Ammonium molybdate spectrophotometer method (B G76 002-90)
13	TN	Potassium persulfate oxidation ultraviolet spectroscopy
14	NH_4_^+^-N	Pay reagent luminosity law
15	DO	YSI 550A dissolved oxygen meters
16	Total Fe	O-Phenanthroline spectrophotometer method
17	Al	HI 93747 NANHA aluminum ion tester
18	Zn	HI 93731 NANHA zinc ion tester
19	Cu	HI 93747 NANHA copper ion tester
20	Me	HI 93748 NANHA manganese ion tester
21	Si	Molybdenum blue colorimetric method
22	Surfactant	Methylene blue spectrophotometer method

**Table 2:** Comparison of water quality of municipal reclaimed water and surface water.

Number	Index	Unit	Municipal reclaimed water	Surface water

1	Turbidity	NTU	1.2~4.3	1.9~5.7
2	pH		7.22~7.91	7.05~8.57
3	Conductivity	*μ*s/cm	930~1101	566~753
4	TDS	mg/mL	540~587	283~356
5	Cl^−^	mg/L	109.53~125.29	26.30~48.45
6	SO_4_^2−^	mg/L	100.15~122.25	84.76~103.29
7	Total alkalinity (CaCO_3_)	mg/L	138.57~143.24	150.25~168.78
8	Total hardness (CaCO_3_)	mg/L	286.36~296.56	268.77
9	Calcium hardness (CaCO_3_)	mg/L	176.5	122.8
10	HCO_3_^2−^	mmol/L	3.464	3.572
11	COD_Mn_	mg/L	4.2~6.8	2.24~3.5
12	TP	mg/L	0.21~0.39	0.13~0.17
13	Soluble TP	mg/L	0.15~0.21	0.10~0.15
14	Orthophosphate	mg/L	0.12~0.17	0.06~0.09
15	TN	mg/L	35.2	23.26
16	Total Fe	mg/L	0.075	0.061
17	Al	mg/L	<0.04	<0.04
18	Zn	mg/L	<0.018	0.12
19	Cu	mg/L	<0.010	<0.010
20	Mn	mg/L	<0.001	0.005
21	Si	mg/L	4.36	1.5
22	Surfactant	mg/L	0.14	<0.05
23	Total bacterial count	cfu/mL	(1.5~4.9) × 10^4^	1.0 × 10^3^
